# Medical students, spirituality and religiosity-results from the multicenter study SBRAME

**DOI:** 10.1186/1472-6920-13-162

**Published:** 2013-12-07

**Authors:** Giancarlo Lucchetti, Leandro Romani de Oliveira, Harold G Koenig, José Roberto Leite, Alessandra LG Lucchetti

**Affiliations:** 1 Federal University of Juiz de Fora, Juiz de Fora, Brazil; 2 Brazilian Medical Spiritist Association, Rua Dona Elisa, 150 apto 153B, São Paulo, Brazil; 3 Federal University of São Paulo, São Paulo, Brazil; 4 Duke University Medical Center, Durham, North Carolina, USA; 5 King Abdulaziz University, Jeddah, Saudi Arabia

**Keywords:** Spirituality, Medical education, Religion and medicine, Curriculum

## Abstract

**Background:**

To evaluate the relationship between spirituality/religiosity (S/R) and the attitudes, beliefs and experiences of medical students in Brazil with respect to S/R in their undergraduate training and clinical practice.

**Methods:**

SBRAME (Spirituality and Brazilian Medical Education) is a multicenter study involving 12 Brazilian medical schools with 5950 medical students (MS). Participants completed a questionnaire that collected information on socio-demographic data and S/R in their undergraduate training and practice.

**Results:**

Of all MS, 3630 participated in the survey (61.0%). The sample was 53.8% women and the mean age was 22.5 years. The majority of MS believed that spirituality has an impact on patients’ health (71.2%) and that this impact was positive (68.2%). The majority also wanted to address S/R in their clinical practice (58.0%) and considered it relevant (75.3%), although nearly one-half (48.7%) felt unprepared to do so. Concerning their training, most MS reported that they had never participated in a “spirituality and health” activity (81.0%) and that their medical instructors had never or rarely addressed this issue (78.3%). The majority also believed that they should be prepared to address spiritual issues related to the health of their patients (61.6%) and that this content should be included in the medical curriculum (62.6%).

**Conclusion:**

There is a large gap between MS attitudes and expectations and the S/R training that they are receiving during their undergraduate training. The majority of MS surveyed believe that patients should have their beliefs addressed and that these beliefs could have important effects on their health and the doctor-patient relationship. These results should stimulate discussion about the place that S/R training should have in the medical curriculum.

## Background

Spirituality/religiosity (S/R) and its relationship to health has been extensively studied during the past few decades [1,2]. Spiritual and religious beliefs have been associated with many aspects of patients’ health and health behaviors, the disease process, medical treatment, medical decisions and physician-patient relationship [3-6].

Within this context, most US and UK medical schools have already included spirituality and health content in their curricula [7,8]. Nonetheless, in a curricula whose content is constrained by time, there are concerns that the inclusion of S/R would take the place of other important topics [8,9].

The main reason for including S/R, according to the Association of American Medical Colleges, is to better understand the role that spirituality plays in patient care, acknowledge the place that it plays in providing compassionate care, appreciate the interaction between biopsychosocial factors and S/R aspects of patients’ lives, and acquire the ability to take a spiritual history [10].

Studies have shown that spiritual issues, while frequently coming up in clinical practice, are seldom addressed by the medical profession [4,11,12]. Lack of training and lack of knowledge are emphasized as important barriers [12,13]. According to recent studies, most physicians and medical educators believe that this issue is important, but few address this issue with their students and residents [12,13].

Many medical students, in contrast, believe that S/R issues have an impact on patients’ health, want to address this issue in their practice, and believe that S/R should be included in the medical curriculum [14,15]. However, many complain that they have not been prepared during their medical training to do so. Studies documenting this, however, have often been regional and the generalizability of these findings have been called into question.

Therefore, studies that include a more representative sample of medical students are important in order to better understand students attitudes and experiences regarding S/R training in medical education.

This multicenter study involving 12 medical schools in Brazil, then, seeks to examine medical students’ attitudes, beliefs, and experiences regarding S/R in their undergraduate training and their clinical practice.

## Methods

### Study design

SBRAME (Spirituality and Brazilian Medical Education) is a cross-sectional, multicenter study involving 12 Brazilian medical schools that have a total enrollment of 5950 medical students (MS) [16]. The study was carried out from June 2010 to September 2011 and was coordinated by the Universidade Federal de São Paulo (UNIFESP), Universidade Federal de Juiz de Fora and Brazilian Medical Spiritist Association, Brazil.

### Medical education in Brazil

Presently, there are 180 medical schools in Brazil. Admission is based on grades obtained on “university entrance exams” which are taken after high school [17]. In order to become a medical doctor, students must complete a full-time program consisting of six years. This includes four years focusing pre-clinical and clinical subjects and two years focusing internship practices at a University Hospital, in which the students work under supervision of residents and staff physicians [15,18].

### Definitions

For the present study, spirituality [2] was considered as “a personal search toward understanding questions about life, its meaning, and its relationships to sacredness or transcendence, that may or may not lead to the development of religious practices or formation of religious communities”. Religiosity [2] was understood as the “extent to which an individual believes, follows, and practices a religion, either organizational (church or temple attendance) or non-organizational (praying, reading books, or watching religious programs on television)”.

### Participating institutions

During a medical congress in Brazil, we invited medical school representatives who were interested in participating in research on “Spirituality and medical education”. From the 14 representatives who attended this meeting, 11 agreed to take part in the study. After this first meeting, one additional medical school decided to participate, resulting in a total of 12 participating schools.

The following medical schools participated in the study: Universidade Federal de São Paulo (UNIFESP)-São Paulo (570 students)-Public (Federal); Faculdade de Medicina de Marília (FAMEMA)-Marília (460 students)-Public (State); Centro Universitário Lusíadas (Lusíadas)-Santos (460 students)-Private; Faculdade de Medicina de São José do Rio Preto (FAMERP)-São José do Rio Preto (370 students)-Public (State); Pontifícia Universidade Católica de Sorocaba (PUC-SP)-Sorocaba (580 students)-Private; Universidade Metropolitana de Santos (UNIMES)-Santos (480 students)-Private; Universidade Nove de Julho (UNINOVE)-São Paulo (580 students)-Private; Faculdade de Ciências Médicas da Santa Casa de São Paulo (Santa Casa)-São Paulo (570 students)-Private; Faculdade de Medicina do ABC (FMABC)-São Paulo (580 students)-Private; Universidade Federal de Santa Maria (UFSM)-Santa Maria (580 students)-Public (Federal); Universidade Federal do Mato Grosso do Sul (UFMS)-Cuiabá (360 students)-Public (Federal) and Faculdade de Medicina de Jundiaí (360 students)-Private.

### Training of researchers

First, a meeting (with at least one member of each medical school) was held to discuss the objectives and assess each institution’s willingness to participate in this study. Next, research supervisors and interviewers from each medical school were trained using a common manual and supplemented by web-based training.

### Procedures and participants’ selection

All MS officially registered in the 12 medical schools were invited by the researchers to take part in the study. MS were personally approached before or after classes and during breaks.

### Data collection instrument

Participants completed a self-administered, multiple choice 43-item questionnaire, which was adapted and expanded from other pilot studies carried out in Brazil [12,15,19] and collected the following information:

•Socio-demographic data: gender, age, family income, ethnicity, religious affiliation and undergraduate year.

•Religiosity: For assessing the religious aspects of participants, the Duke Religious Index (DUREL) was used [20]. DUREL is a five-item measure of religious involvement made up of three subscales: (1) organizational religious behavior - religious attendance (1 item), (2) non-organizational religious behavior - praying, scripture reading, meditation, among others (1 item), and (3) intrinsic religious motivation (3 items). Response options are on a 5- or 6-point Likert scale. It has been validated in Portuguese by Lucchetti et al. [21].

•Spirituality: the concept of spirituality was evaluated using a close-ended question with the following five response options (checking more than one option was possible): “Belief and relationship with God/religiosity”, “Search for meaning and significance for human life”; “Belief in the existence of soul and life after death”; “Belief in something that transcends matter”; and “Ethical and humanistic posture”.

•Clinical practice: this section asked questions about the connection between spirituality and health in clinical practice, including the influence of spirituality in patients’ health and in the patient-physician relationship, MS preparedness and willingness to address S/R, barriers to addressing S/R, whether MS had ever asked a patient about S/R, and whether it is appropriate for a doctor to pray with a patient.

•Undergraduate education: this section asked about the role of Brazilian medical schools and medical faculty in providing training on the topic of S/R. Questions covered whether medical faculty had ever addressed S/R in the curriculum, whether MS should be prepared for this approach, whether Brazilian medical schools were giving all required information in this field, how MS sought knowledge for addressing S/R, how medical schools should address this issue, and whether the MS’s spirituality had changed since their entrance into medical school.

### Statistical analysis

Data were entered into an Excel database and analyzed using Statistical Package for Social Sciences program (SPSS), version 17.0. Descriptive statistics were used to describe the range of responses. For categorical variables, the descriptive statistics are reported as numbers and percentages. For continuous data, the descriptive statistics include mean and standard deviations. For all non-normally distributed data, the median was given along with the mean and standard deviation. All confidence intervals were 95%.

### Ethical issues

Participants gave written informed consent and the study was approved by the ethics committees of Universidade Federal de São Paulo and the other medical schools.

## Results

### Sample

A total of 3630 medical students participated in the study (61.0% response rate). The most common reasons for not participating were: refused, did not wish to sign the consent form, were interviewers in the study, or were absent at the time of the survey.

### Socio-demographic characteristics

A slight majority of MS were women (53.8%), mean age was 22.5 years (SD: 4.6) and median was 22 years, 82.2% were white, and 51.1% had an average family income of more than US$4000 per month (51.1%). Participants were distributed across the following medical school years: 612 (16.9%) in the first year; 752 (20.7%) in the second year; 559 (15.4%) in the third year; 635 (17.5%) in the fourth year; 579 (15.9%) in the fifth year, and 485 (13.6%) in the sixth year.

### Institutions’ characteristics

There were 7 (58.3%) private schools and 5 (41.7%) public schools (3 federal and 2 state) and most institutions (83.3%) were from South-east Brazil (the most wealthy region in Brazil).

### Medical students’ spiritual and religious beliefs

Most MS (66.1%) had a religious affiliation (Catholics followed by Spiritists); believed in God (84.2%); attended religious services less than once a week (81.8%); spent more than once a week in private religious activities (55.8%) and believed that the human body included a soul (78.9%). The mean of DUREL intrinsic religiosity was 9.63 (SD: 3.69) and the median was 10, ranging from 3 (low intrinsic religiosity) to 15 (high intrinsic religiosity) (Table 1).

**Table 1:** Medical students’ religious beliefs

**Spiritual/religious beliefs**	**n**	**%**
Religious affiliation		
No religion and not believe in God	300	8.3
No religion, but believe in God	923	25.6
Catholics	1245	34.6
Protestant Evangelicals	291	8.0
Spiritists	470	13.0
Others	370	10.5
How often do you attend church or other religious meetings?		
Once a week or more	649	18.2
Less than once a week and more than once a year	1628	45.2
Once a year or less/Never	1320	36.6
How often do you spend time in private religious activities (prayer, Bible study, etc.?)		
Daily or more	1173	32.7
Less than daily and at least once a week	829	23.1
Less than once a week/Never	1597	44.2
In my life, I experience the presence of the Divine (i.e., God)		
Definitely true of me or Tends to be true	2379	66.3
Unsure/Tends not to be true/Definitely not true	1207	33.7
My religious beliefs are what really lie behind my whole approach to life.		
Definitely true of me/Tends to be true	1772	49.3
Unsure/Tends not to be true/Definitely not true	1821	50.7
I try hard to carry my religion over into all other dealings in life.		
Definitely true of me/Tends to be true	1387	38.6
Unsure/Tends not to be true/Definitely not true	2202	61.4
Do you believe in God?		
Yes	3020	84.2
No	316	8.8
No opinion	251	7.0
Do you believe that after death, the soul/spirit remains alive?		
Yes	2395	66.8
No	520	14.5
No opinion	669	18.7
Do you believe the human body is composed by a body and a soul?		
Yes	2841	78.9
No	336	9.3
No opinion	421	11.8
Do you believe in reincarnation		
Yes	1319	36.9
No	1444	40.4
No opinion	806	22.7

### Understanding of spirituality

The most common understanding of spirituality was “belief and relationship with God/religiosity” (38.8%), followed by “search for meaning and significance for human life” (38.0%), and by “belief in the existence of the soul and life after death” (20.5%) (Table 2).

**Table 2:** Medical students’ opinions concerning spirituality, religiosity and health

**Medical students’ opinions**	**n**	**%**
How do you define spirituality? (checking more than one alternative was possible)		
Belief and relationship with God/Religiosity	1408	38.8
Search for meaning and significance for human life	1380	38.0
Belief in the existence of soul and life after death	747	20.5
Belief in something that transcends matter	1555	42.8
Ethical and humanistic posture	684	18.8
Does spirituality influence the health of patients?		
A lot	2568	71.2
Somewhat	852	23.7
A little or no influence	187	5.1
Is this influence positive or negative?		
Positive	2458	68.2
Negative	170	4.7
Positive and negative	917	25.4
No influence at all	54	1.7
Influence on patient-physician relationship and health disease process		
High influence	1328	36.8
Moderate Influence	1368	37.9
Little or no influence	910	25.3
Do you want to address your patients’ religious/spiritual issues?		
Yes	2073	58.0
No	1500	42.0
Do you feel prepared to address it?		
A lot	358	10.5
Somewhat	1386	40.8
A little or no prepared	1645	48.7
How relevant is the approach of spirituality for a doctor?		
A lot	1102	30.6
Somewhat	1608	44.7
A little or not relevant	887	24.6
Is it appropriate for a doctor to pray with a patient?		
Never	818	22.8
Only if the patient asks for	2250	62.7
Every time the doctor think it is appropriate	516	14.3

### Clinical practice

The majority of MS indicated that spirituality had an impact on the health of patients (71.2%), that this impact was positive (68.2%), and that addressing spiritual issues was relevant (75.3%). The majority of MS wanted to address these issues (58.0%), but nearly half said they were not prepared to do so (48.7%) (Table 2).

With regards to their clinical practice, 64.1% had already addressed this issue with patients, but usually did not ask about S/R on a routine basis (54.9%). Most MS believed that patients do not feel uncomfortable when asked about S/R issues (80.6%) and that the most common reasons for not addressing S/R were: “fear of imposing religious beliefs” (47.5%), “fear of offending patients” (35.8%), and “lack of knowledge” (34.7%). The most frequent S/R treatments recommended to their patients were: prayer (67.7%), scripture reading (31.5%) and volunteering in religious communities (18.8%) (Table 3).

**Table 3:** Clinical practice and spirituality/religiosity

**Medical students’ opinions and practice**	**n**	**%**
Have you ever addressed patients’ spiritual/religious beliefs?*		
Yes	1805	64.1
No	1010	35.9
How often do you ask spiritual/ religious issues with your patients?*		
I do not ask	1010	35.9
Rarely	536	19.0
Sometimes	615	21.8
Often	654	23.2
Do patients feel uncomfortable when asked about spiritual/religious issues?*		
I do not ask	1010	-
Rarely	1455	80.6
Sometimes	464	16.6
Often	50	2.7
Which barriers discourage you to discuss religion/spirituality with patients?*		
Lack of Knowledge	1249	34.7
Lack of training	1113	30.8
Lack of time	1006	27.9
Uncomfortable with this issue	726	20.1
Fear of imposing religious beliefs	1713	47.5
Religion/spirituality are not relevant for medical treatment	279	7.7
It’s not my job	376	10.4
Fear of offending the patients	1292	35.8
Disapproval of my colleagues	232	6.4
Which religious treatments would you recommend to your patients?		
Prayer	2390	67.7
Bible/scripture/religious literature	1130	31.5
Fluidic water/Energized Water/Holy Water	330	9.2
Disobsession/Exorcism	128	3.5
Hands imposition/Reike/Johrei/Spiritist passé	432	12.0
Charity in religious communities	677	18.8

*734 MS students were excluded from this question because they have never seen patients.

### Education

Most MS reported that they had never received any training during medical school on “spirituality and health” (81.0%) and that medical faculty had never or only rarely addressed this issue (78.3%). The majority believed that they should be prepared to address spirituality with their patients (61.6%) and that Brazilian medical schools were not providing adequate training in this area (83.4%). Many students felt that content on S/R should be included in the medical curriculum (62.6%), and about half preferred that it be elective (47.8%) (Table 4).

**Table 4:** Academic education and the subject ‘spirituality’

**Medical students’ opinions**	**n**	**%**
Have medical teachers ever addressed this issue with you?		
Never	1541	43.8
Rarely	1212	34.5
Sometimes	697	19.8
Frequently	63	1.9
Are Brazilian medical schools giving all required information in this field?*		
A little or no	2811	83.4
Somewhat	446	13.2
Yes, a lot	104	3.4
Should MS be prepared to address this issue?**		
A little or no	1334	38.3
Somewhat	947	27.1
Yes, a lot	1202	34.5
Have you ever taken part in a “Spirituality and health” activity?		
Yes	686	19.0
No, but I would like to	1947	54.1
No and I would not like to	964	26.9
Should “Spirituality and health” be included in medical curricula?		
Yes	2219	62.6
No	1324	37.4
How should this issue be addressed in the medical curricula?***		
Compulsory discipline	174	5.4
Elective discipline	1542	47.8
Integrated with other disciplines	747	23.3
Through events, courses and stage	757	23.5
How do you seek knowledge about “health and spirituality” issues? (checking more than one alternative was possible)		
I do not seek	1537	42.5
Lectures and events	443	12.2
Books related to this issue	777	21.5
Scientific articles	331	9.1
With medical teachers from my university	244	6.7
Within my religion	1135	31.4
Have your beliefs changed after your entrance into university?		
Yes	1022	28.6
No	2547	71.3
Is your experience as a medical student responsible for this change?		
Yes	563	55.0
No	459	45.0

*186 MS have no opinion regarding this question.

**126 MS have no opinion regarding this question.

***341 MS have no opinion regarding this question.

MS tended not to seek out knowledge about “health and spirituality” (42.5%), and of those who did, they sought this knowledge within their own religion (31.4%). Few MS sought out knowledge on S/R from scientific articles (9.1%). Most had not changed their S/R beliefs since entrance into medical school (71.3%) (Table 4).

## Discussion

This multicenter study advances our understanding of the place that training on spirituality/religion and health should have in medical education. Many of these Brazilian medical students believed that spirituality had an influence on patients’ health and wanted to address this in clinical practice. Nevertheless, the majority felt they were not prepared to do so and that medical school was not providing the necessary training. These results suggest that there is a gap between students’ attitudes/needs in this area and the training they are getting.

Other studies have reported similar findings among practicing physicians [11,22-25], medical school faculty [12], and medical students in other settings [15].

Monroe et al. [11] found that 85% of physicians agreed that they should be aware of a patient’s religious and spiritual beliefs, although few addressed it on a routine basis. Curlin et al. [22] also reported that most physicians believed that S/R had an influence on health and that S/R helped patients to cope. Some studies, however, find that less than 20% of physicians regularly discuss spiritual topics in patient encounters [4,23-26].

Likewise, Mariotti et al. [12] found that more than 72% of medical faculty believed that faith or spirituality can positively influence the treatment of patients, while only 43.4% reported they felt prepared to do so.

With regards to medical students, Banin et al. [15] found that most MS believed that spirituality had an impact on patient’s health, but again few students felt prepared to address spiritual issues in clinical practice.

In the present study, the most common reasons for not addressing these issues were “fear of imposing religious beliefs”, “fear of offending patients” and “lack of knowledge” on how to address S/R issues. These barriers could be overcome by training. Studies [12,27,28] in practicing physicians have also found that most common barriers to addressing S/R issues with patients are lack of time, lack of knowledge, and lack of training. MS in the present study, however, appeared more worried about causing harm to patients than they were about the time spent in addressing S/R issues.

Another important finding was the lack of training that MS received on spirituality and health. Most students had received no training on this subject and believed that Brazilian medical schools were not adequately preparing to address S/R issues in clinical practice. These findings are consistent with a study by Rasinski et al. [29] in the U.S. who reported that only 23% of practicing physicians received formal training in this area. Likewise, Mariotti et al. [12] found that 92.3% of medical faculty felt that medical schools were not providing adequate training in this area. The majority of students in the present study believed that S/R should be included in the medical curriculum.

More than 40% of students reported they sought information about S/R and health through a religious source, rather than through scientific papers, other academic medical publications, or their medical professors, which is similar to what has been found in nursing students [19].

These findings reflect in part MS’s fear of imposing their own beliefs on patients and the difficulties in discriminating religiosity from spirituality. We note that when asked about their understanding of spirituality, most choose “belief and relationship with God/religiosity” which is more associated with the concept of religiosity [2]. The differences between spirituality and religiosity should be defined for medical students to help clarify the differences between these two dimensions.

Concerning the religious beliefs of MS themselves, 66.1% had a religious affiliation and 18.2% attended religious services frequently. This level of involvement in S/R is different than that found in the general population of Brazil in which 95% have a religious affiliation and 37% frequently attend religious services [30]. However, it is consistent with a previous study of physicians in the Northeastern U.S. [31]. Despite not having a religious affiliation, however, many MS reported belief in God.

Most MS in this study felt that S/R issues had an impact on patients’ health and many believed that it should be included in the medical curriculum, which is consistent with the goal of spirituality and health courses proposed in the U.S. by Puchalski [32], which include “An understanding that the spiritual dimension of people’s lives gives an avenue for compassionate caregiving” and “the ability to apply the understanding of a patient’s spiritual and cultural beliefs and behaviors to appropriate clinical contexts”.

Understanding the complex relationship between S/R, medical practice, and medical education could open new perspectives for a different, more compassionate and more integrated approach to patient patient care. Findings from studies such as the present one may contribute to the discussion of the role of S/R in medical curricula, and perhaps to help shift medical education into a more bio-psycho-socio-spiritual model.

The present study has limitations that affect its applicability. First, the study was carried out in Brazil and data should be replicated in other cultural contexts. Second, the response rate was 61% and does not include the opinions of over one-third of students who may have had less interest in the topic and so refused to participate. However, this response rate is similar to or better than other multicenter studies in medical education [33-36]. Third, only 12 of 180 Brazilian medical schools participated in the present study. Therefore, readers should be cautious in generalizing findings to all medical students in Brazil. Fourth, the differences between responders and non-responders and between the institutions who did and did not participate in the survey were not assessed. Fifth, possible variations in the makeup of students attending each institution may also exist, particularly between private and public medical schools.

The present study also has a number of strengths. This is one of the most comprehensive studies of the attitudes and experiences of medical students [33,34,36-38] and, to our knowledge, the largest study to date dealing with S/R in medical education [39].

## Conclusions

In conclusion, there is a gap between MS attitudes and expectations about the inclusion of S/R in their training and clinical practice. Many Brazilian MS feel that patients should have their beliefs addressed and that these beliefs could have important impact on medical outcomes and patient-physician relationship. The results of this study could foster a more open discussion of the place that spirituality should take in the medical curriculum in order to achieve a more patient-centered medicine.

## Competing interests

The authors declare they have no competing interests.

## Authors’ contributions

GL, JRL and ALGL, LRO participated in the design of the study. GL coordinated collection of medical students’ questionnaires. SBRAME Collaborators applied the questionnaires. GL performed the statistical analyses. GL, JRL HGK, ALGL, LRO interpreted the data. GL, HGK and ALGL drafted the first version of the manuscript. All authors critically revised the manuscript. All authors read and approved the final manuscript.

## Pre-publication history

The pre-publication history for this paper can be accessed here:

http://www.biomedcentral.com/1472-6920/13/162/prepub

## SBRAME collaborators

Adriane Rodrigues, Adriano Barbosa, Amanda Vanzella, André Perdicaris, Bruno Borges, Camila Theodoro, Daniel Moreira, Daniela Martins, Daniela Paraskevopoulos, Daniele Espinha, Danielle Barros, Danilo Pereira, Denise Horly, Diego Borges, Diego Carmo, Edson Tamaki, Eduardo Torres, Eliza Cecheti, Everton Crivelaro, Fabiola Cavalieri, Fátima Scattolin, Fernanda Bacaro, Fernanda Albuquerque, Gabriela Longo, Gabriela de Oliveira, Giani Cioccari, Gilberto Luppi, Gilmor Farenzena, Helena Fonseca, Janaina Evangelista, Jean Zeferino, Jéssica Parreira, José Fittipaldi Neto, José Aldrighi, Juliana Santana, Juliana Silva, Julio Milanezi Neto, Lazslo Ávila, Lessanie Rodrigues, Lidia Gonçalves, Luciana Valdez, Mariana Salvi, Mariana Mierel, Mario Peres, Maurício Gonçalves, Michelle Yakabe, Nicolle Taissun, Patrícia Novaes, Rafael Silva, Rafael Latorraca, Raphael da Silva, Raquel de Oliveira, Renato Barros, Roberto Topolniak, Rodolpho da Costa, Sara Progiante, Sonia de Andrade, Sthephanie de Camargo, Tassia Serra, Túlio Soares, Victor Schnor.
